# FAK is a Critical Regulator of Neuroblastoma Liver Metastasis

**DOI:** 10.18632/oncotarget.732

**Published:** 2012-11-16

**Authors:** Sora Lee, Jingbo Qiao, Pritha Paul, Kathleen L. O'Connor, B. Mark Evers, Dai H. Chung

**Affiliations:** ^1^ Departments of Pediatric Surgery, Vanderbilt University Medical Center1, Nashville, TN; ^2^ Cancer Biology, Vanderbilt University Medical Center1, Nashville, TN; ^3^ Department of Surgery, University of Kentucky, Lexington, KY; ^4^ Markey Cancer Center, University of Kentucky, Lexington, KY

**Keywords:** GRP-R, FAK, malignant transformation, metastasis, neuroblastoma

## Abstract

Neuroblastomas express increased levels of gastrin-releasing peptide receptor (GRP-R). However, the exact molecular mechanisms involved in GRP-R-mediated cell signaling in neuroblastoma growth and metastasis are unknown. Here, we report that focal adhesion kinase (FAK), as a critical downstream target of GRP-R, is an important regulator of neuroblastoma tumorigenicity. We found that FAK expression correlates with GRP-R expression in human neuroblastoma sections and cell lines. GRP-R overexpression in SK-N-SH cells increased FAK, integrin α3 and β1 expressions and cell migration. These cells demonstrated flatter cell morphology with broad lamellae, in which intense FAK expression was localized to the leading edges of lamellipodia. Interestingly, FAK activation was, in part, dependent on integrin α3 and β1 expression. Conversely, GRP-R silencing decreased FAK as well as Mycn levels in BE(2)-C cells, which displayed a denser cellular morphology. Importantly, rescue experiments in GRP-R silenced BE(2)-C cells showed FAK overexpression significantly enhanced cell viability and soft agar colony formation; similarly, FAK overexpression in SK-N-SH cells also resulted in increased cell growth. These effects were reversed in FAK silenced BE(2)-C cells *in vitro* as well as *in vivo*. Moreover, we evaluated the effect of FAK inhibition *in vivo*. FAK inhibitor (Y15) suppressed GRP-induced neuroblastoma growth and metastasis. Our results indicate that FAK is a critical downstream regulator of GRP-R, which mediates tumorigenesis and metastasis in neuroblastoma.

## INTRODUCTION

Neuroblastoma is highly aggressive with frequent metastases, which contributes to overall significant morbidity and mortality [1]. We have shown that gastrin-releasing peptide receptor (GRP-R), a G-protein coupled receptor, is involved in neuroblastoma cell survival, invasive potential and metastasis [2, 3]. We reported that the upregulation of GRP-R increases the binding capacity for its ligand GRP, resulting in a faster constitutive neuroblastoma cellular growth rate [2]. Conversely, downregulation of GRP-R reversed the aggressive cell phenotype and inhibited liver metastases *in vivo* [3]. Therefore, GRP-R-mediated signaling plays critical roles in tumorigenesis and metastasis in neuroblastoma. However, we have yet to clearly define the molecular mechanisms responsible for GRP-R-mediated tumorigenicity.

Focal adhesion kinase (FAK), a 125-kDa cytoplasmic non-receptor protein tyrosine kinase, plays an essential role in cell adhesion and migration [4]. FAK is comprised of a central catalytic domain flanked by large N- and C-terminal non-catalytic domains. The N-terminal domain of FAK binds to sequences in the cytoplasmic domain of β-integrin subunits, thereby functioning as an important member of the integrin signaling pathway. The C-terminal region of FAK is rich in protein-protein interaction sites, directing FAK to newly-formed and existing adhesion complexes [4]. Cancers are known to express FAK, which is responsible for stimulated cell motility, invasiveness and proliferation [5-7]. FAK activation is involved in various intracellular pathways, including GRP-mediated cell signaling [8, 9].

High levels of GRP-R and FAK have been reported in prostatic tissues from patients with advanced cancer and in tumorigenic cell lines [5]. One report showed that expression of FAK and phosphorylated (p)-FAK (Y397) correlates with the degree of colon cancer cell differentiation as well as to GRP/GRP-R co-expression [10]. Bombesin (BBS), an amphibian equivalent of GRP, induces PC-3 cell motility through FAK activation [11]. We and others have shown that GRP and BBS bind to GRP-R with high affinity to stimulate neuroblastoma cell growth in an autocrine and/or paracrine fashion [11, 12]. However, the intracellular signaling mechanisms involved in GRP/GRP-R-mediated FAK activation and subsequent neuroblastoma cell growth, motility and metastasis remain unclear.

In this study, we show that GRP-R and FAK expressions in human neuroblastoma tissues and cell lines correlate with tumor malignancy. Exogenous GRP induced FAK activation at Y397 and enhanced cell migration. Interestingly, GRP-R overexpression increased FAK, integrin expressions as well as cell migration in SK-N-SH cells. Conversely, GRP-R silencing resulted in decreased FAK and Mycn proteins in BE(2)-C cells while FAK overexpression in GRP-R silenced BE(2)-C cells rescued cell growth. Moreover, FAK overexpression alone led to an increase in soft agar colony formation in SK-N-SH cells, whereas FAK silencing resulted in decreased colony formation in BE(2)-C cells. We also found that FAK silencing in BE(2)-C cells suppressed tumorigenesis and metastasis *in vivo.* Furthermore, using an intrasplenic murine model and bioluminescence imaging system, we confirmed that treatment with Y15, a FAK inhibitor, blocks BBS-induced neuroblastoma growth and liver metastases *in vivo*. Our results demonstrated that FAK correlates with GRP-R, and that it exerts oncogenic effects in neuroblastoma as a mediator of GRP-R signaling pathway. Hence, FAK may be a clinically important therapeutic target in the treatment of neuroblastomas.

## RESULTS

### GRP-R and FAK correlated with malignant potential of neuroblastoma, and GRP induced FAK activation (Y397) and cell migration

**Figure 1:**
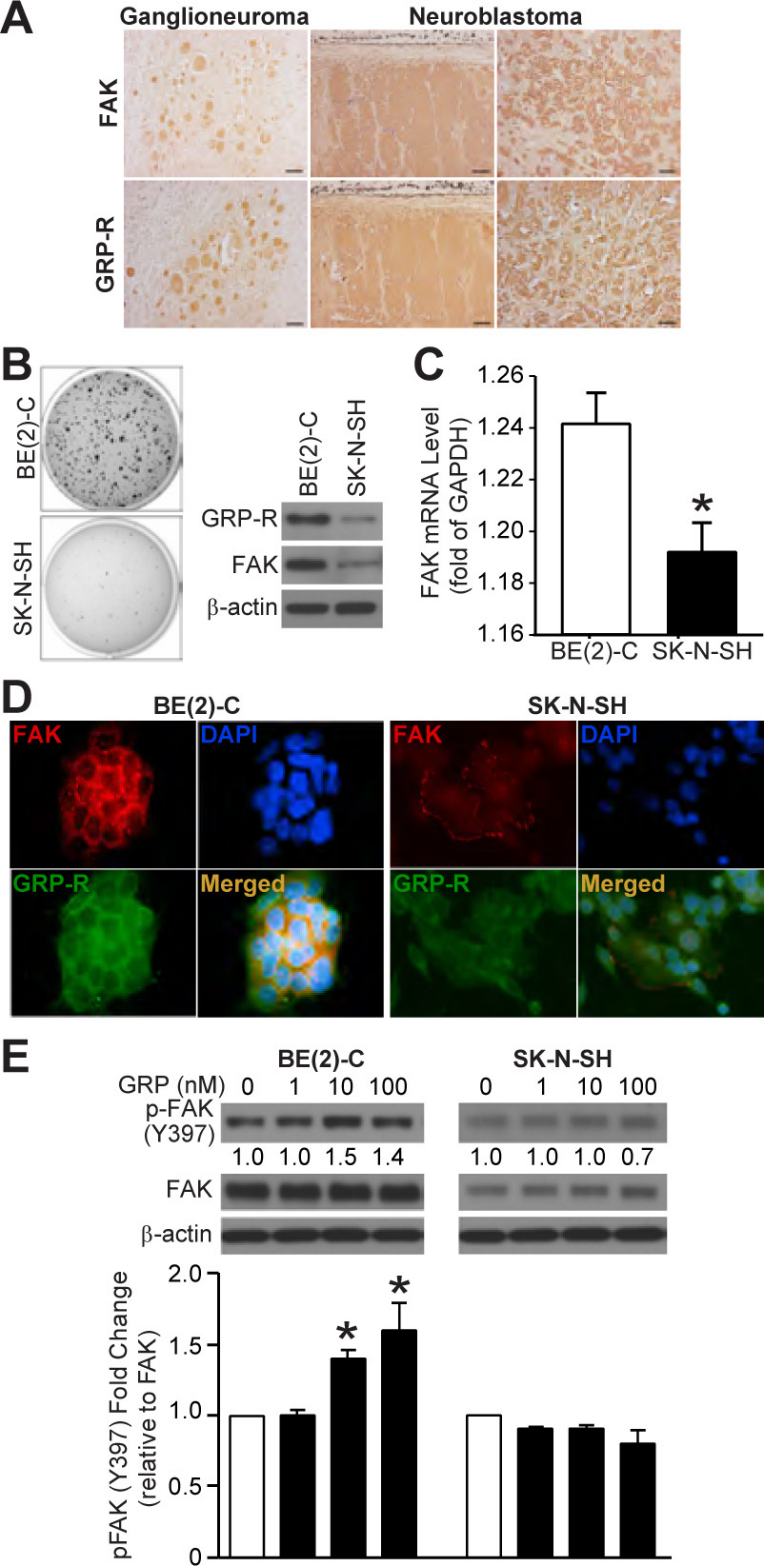
GRP-R and FAK expressions correlate to neuroblastoma malignancy and GRP-induced FAK activation (Y397) (A) Representative histological sections from human ganglioneuroma and undifferentiated neuroblastomas showed similar FAK (*top row*) and GRP-R (*bottom row*) expressions by immunohistochemistry (100× magnification, 20 μm bar). (B) Increased number of colony formation was observed in BE(2)-C cells. BE(2)-C cells demonstrated higher constitutive GRP-R and FAK protein levels when compared to SK-N-SH cells by immunoblotting. β-actin showed relatively equal loading. (C) A higher basal level of FAK mRNA was also observed in BE(2)-C cells. FAK mRNA level was expressed as relative copies of FAK/GAPDH (*= *p* <0.05 vs. BE(2)-C). (D) More intense immunofluorescence of FAK and GRP-R were observed in BE(2)-C cells when compared to SK-N-SH cells (600× magnification). (E) Exogenous GRP for 5 min after overnight serum starvation increased p-FAK (Y397) as measured by immunoblotting; increased p-FAK by 10 nM and 100 nM of GRP in BE(2)-C was observed when compared to SK-N-SH cells (*= *p* <0.05 vs. no treated control).

We reported that an increased GRP and GRP-R expression is found in more undifferentiated neuroblastoma [12]. FAK expression has been correlated with advanced-stage neuroblastoma [13]. In this study, we wanted to determine whether GRP-R expression is associated with FAK in neuroblastoma. First, we performed immunohistochemistry to assess GRP-R and FAK expression in seven paraffin-embedded tumor sections consisting of five undifferentiated neuroblastomas and two ganglioneuromas. As expected, increased FAK expression was noted in undifferentiated neuroblastomas when compared to more benign phenotype of ganglioneuromas; its expression also correlated with GRP-R (Fig. 1A). But due to the limited sample size, we could not determine a correlation of these two protein markers with clinical disease staging. Next, when grown on soft agar, BE(2)-C cells exhibited significantly more colony formation (Fig. 1B), which indicates malignant potential. Consistent with GRP-R protein levels, we found higher levels of FAK protein (Fig. 1B) as well as mRNA (Fig. 1C) in BE(2)-C when compared to SK-N-SH cells. Immunofluorescence also demonstrated that BE(2)-C cells show more intense GRP-R and FAK expression when compared to SK-N-SH cells (Fig. 1D). FAK activation at Y397 site by GRP is well established in 293 HEK cells and various other cancer types [8, 11, 14]. When GRP was exogenously administered for 5 min, we noted a dose-dependent increase in p-FAK levels in BE(2)-C but not SK-N-SH cells (Fig. 1E). Moreover, we also found that GRP treatment induces significant BE(2)-C cell migration in both transwell migration (Supplemental data; Fig. S1B). These results show that FAK expression correlates with malignant potential induced by increased GRP-R expression in neuroblastoma. Our results also support for the important role of FAK as a mediator of GRP/GRP-R signaling, and further validate GRP as an inducer of cell migration in neuroblastoma.

### GRP-R overexpression increased FAK and cell migration in SK-N-SH

**Figure 2:**
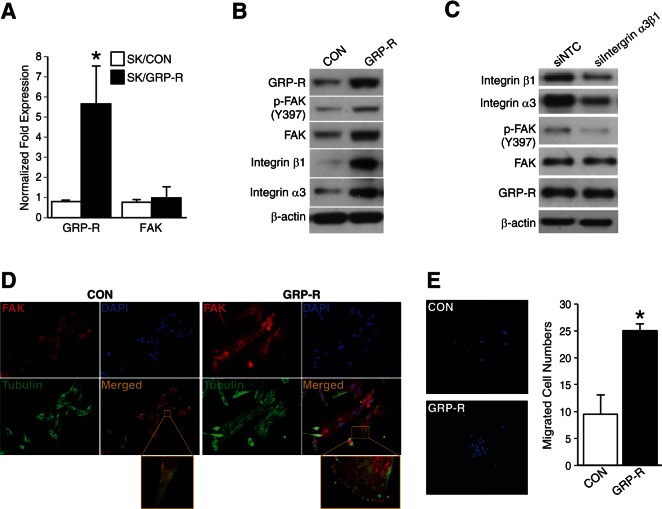
GRP-R overexpression increased FAK and cell migration in SK-N-SH cells (A) Increased levels of GRP-R mRNA but no significant increase of FAK mRNA were observed in GRP-R overexpressing SK-N-SH cells (*= *p* <0.05 vs. SK/CON). (B) Increased FAK expression as well as integrin α3 and β1 levels in GRP-R overexpressing SK-N-SH cells were confirmed by immunoblotting. β-actin demonstrated relatively equal loading. (C) Dual silencing of integrin α3 and β1 (siIntegrin α3β1) in GRP-R overexpressing SK-N-SH cells decreased expression of p-FAK (Y397). β-actin demonstrated relatively equal loading. (D) GRP-R overexpressing SK-N-SH cells demonstrated altered cellular morphology to a flatter appearance along with significantly more intense FAK immunofluorescence (*red color*). Merged image indicates FAK, Tubulin (*green color*) and nuclei (400× magnification). A representative higher magnification image (*enlarged box*) showed intense FAK localization at the leading edges. (E) GRP-R overexpressing SK-N-SH cells migrated more in collagen type I-coated transwell plates when compared to the control. Representative images of DAPI staining for counting were shown (200× magnification) (*= *p* <0.05 vs. CON).

To determine the positive relationship between FAK and GRP-R in neuroblastoma cells, we next performed studies using a GRP-R overexpressing SK-N-SH cell line established in our laboratory [2]. In figure 2A, GRP-R overexpressing SK-N-SH cells showed increased GRP-R mRNA expression but no significant increase of FAK mRNA (*= *p* <0.05 vs. SK/CON). However, immunoblotting showed that FAK protein is upregulated in GRP-R overexpressing SK-N-SH cells when compared to controls (Fig. 2B). Interestingly, integrin α3 and β1 expressions were also significantly upregulated in GRP-R overexpressing cells (Fig. 2B). To confirm whether increased FAK activation in GRP-R overexpressing cells is dependent on these upregualted integrin expressions, we next performed dual silencing of integrin α3 and β1 (siIntegrin α3β1) in GRP-R overexpressing SK-N-SH cells and found that siIntegrin α3β1 significantly decreased p-FAK expression (Fig. 2C). Additionally, to validate these findings and to localize FAK expression, we next performed immunofluorescence study. GRP-R overexpressing SK-N-SH cells, which have an altered cell morphology exhibiting a flatter shape with broad lamellipodial projections, showed significantly enhanced FAK expression at the leading edges of cells (Fig. 2D). Furthermore, GRP-R overexpressing SK-N-SH cells exhibited increased cell migration in the transwell plates coated with collagen type I (Fig. 2E). These results indicate that GRP-R regulates FAK levels post-transcriptionally and FAK activation is regulated in part by integrin expressions in GRP-R overexpressing SK-N-SH cells.

### GRP-R silencing decreased FAK and cell migration in BE(2)-C cells

**Figure 3:**
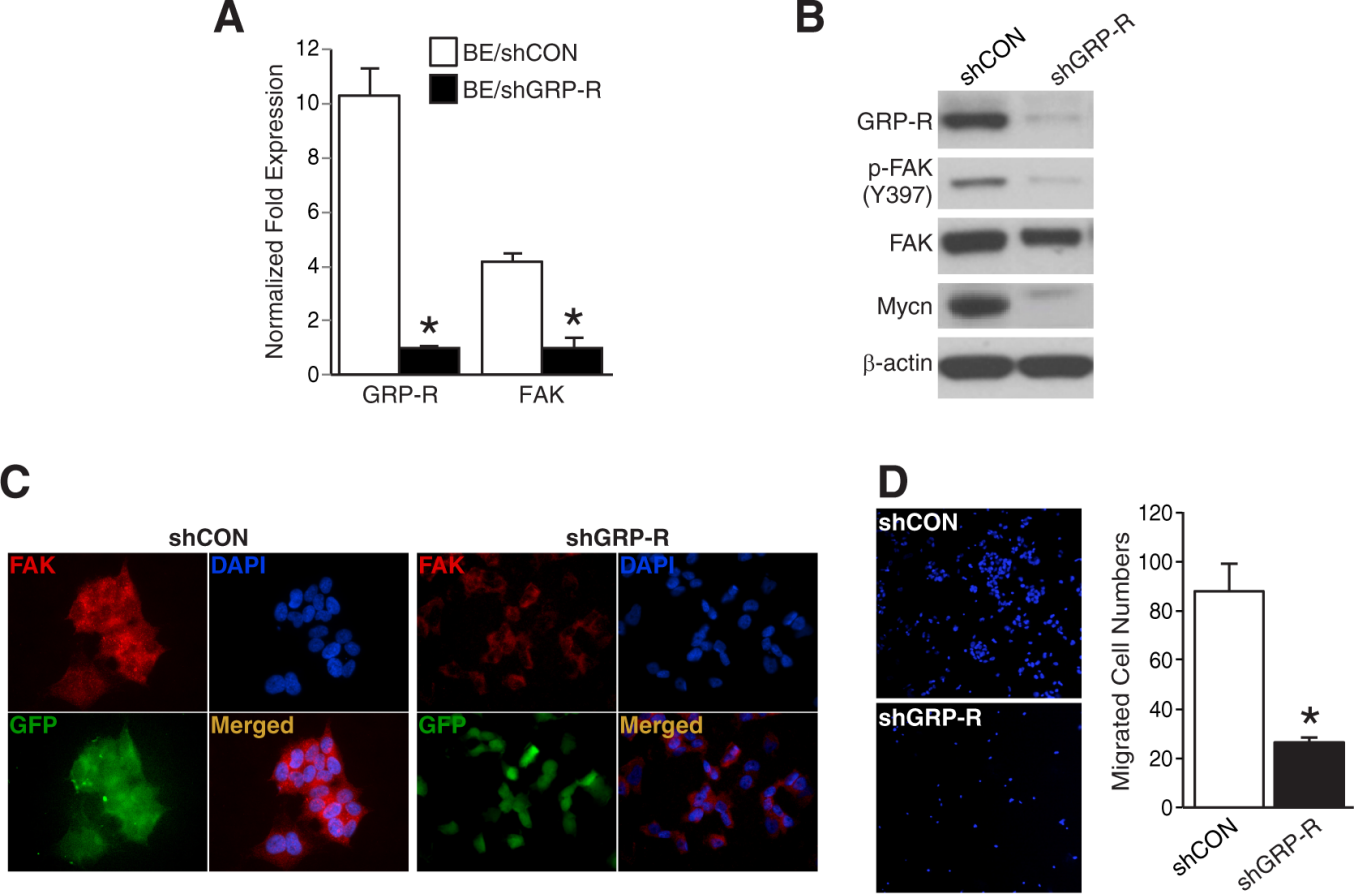
GRP-R silencing decreased FAK and cell migration in BE(2)-C cells (A) Decreases in both GRP-R mRNA as well as FAK mRNA expression were in GRP-R silenced BE(2)-C cells (*= *p* <0.05 vs. BE/shCON). (B) Decreased FAK and Mycn expressions in GRP-R silenced BE(2)-C cells (shGRP-R) was confirmed by immunoblotting. β-actin demonstrated relatively equal loading. (C) shGRP-R demonstrated a rounder, smaller morphology and showed weaker FAK immunofluorescence (*red color*) when compared to control cells (shCON). Merged image indicates FAK and nuclei (400× magnification). (D) shGRP-R migrated less in collagen type I-coated transwell plates when compared to shCON. Representative images of DAPI staining for counting were shown (200× magnification) (*= *p* <0.05 vs. shCON).

Next, to further validate the correlation between GRP-R and FAK, we used stably-transfected GRP-R silenced BE(2)-C cells (shGRP-R) established in our laboratory [3]. In figure 3A, GRP-R silenced BE(2)-C cells showed decreases in both GRP-R as well as FAK mRNA levels (*= *p* <0.05 vs. BE/shCON). Furthermore, we found that both phosphorylated and total FAK protein levels were decreased in shGRP-R (Fig. 3B). Interestingly, we also found that shGRP-R cells downregulated Mycn (Fig. 3B), which is a well known transcription factor of FAK in neuroblastoma [15, 16]. Consistent with these findings, immunofluorescence showed significantly weaker FAK expression in shGRP-R when compared to control cells (shCON) (Fig. 3C). Moreover, the appearance of BE(2)-C cells changed from their typical flat and aggregated morphology to a small and rounder shape after transfection with silencing of GRP-R. Furthermore, shGRP-R cells exhibited decreased cell migration in the transwell plates coated with collagen type I (Fig. 3D). Hence, our results support a positive correlation between GRP-R and FAK, indicating that GRP-R is important in the regulation of FAK-induced changes in cell morphology and increased migration in neuroblastoma cells.

### FAK regulated neuroblastoma cell growth in vitro and in vivo

**Figure 4:**
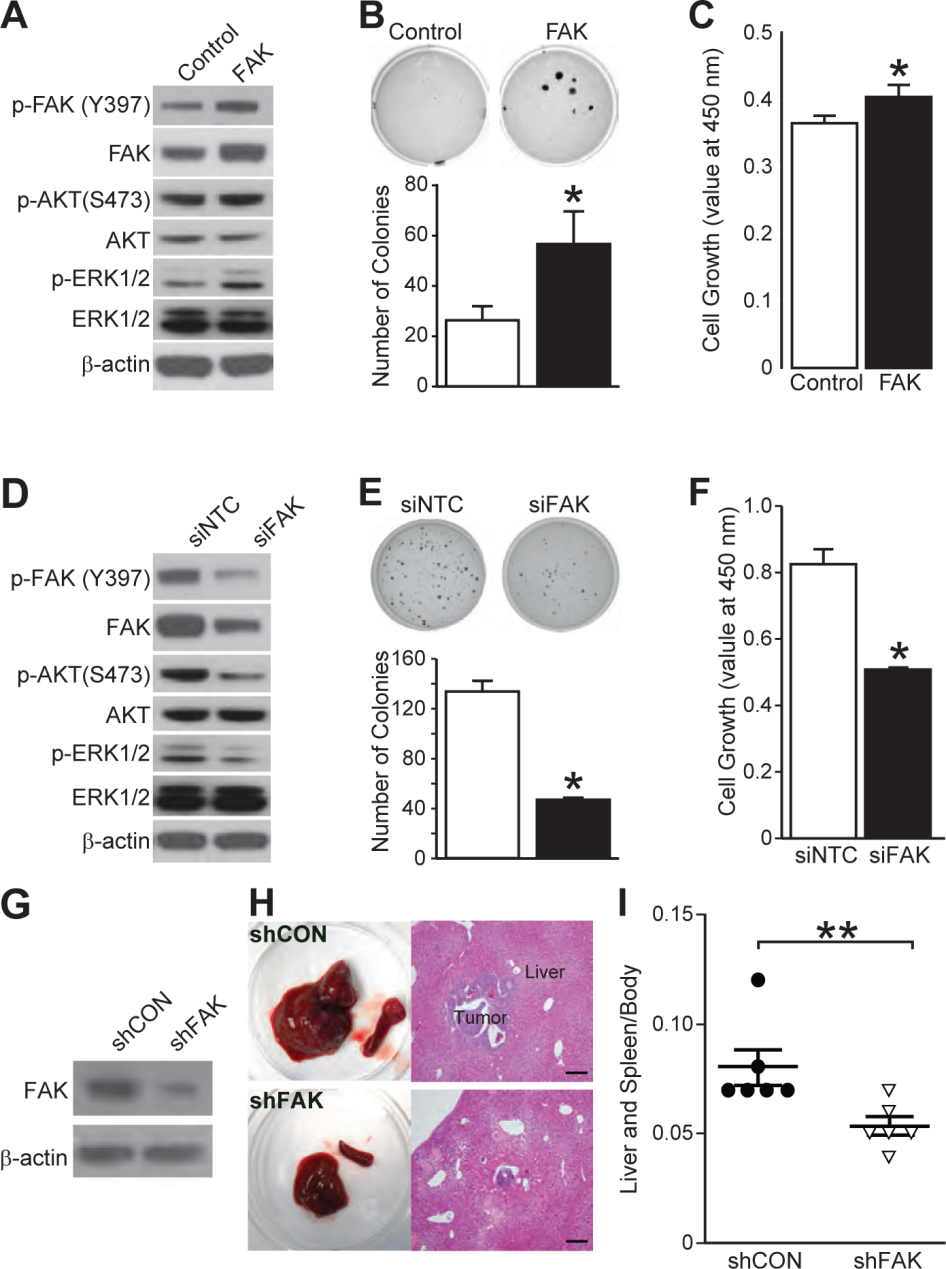
FAK regulates neuroblastoma cell growth *in vitro* and *in vivo* (A) Immunoblotting demonstrated FAK overexpression in SK-N-SH cells. pCMV6-XL4 was used for control. Phosphorylated and total protein levels of AKT and ERK1/2 were tested in the cells. β-actin demonstrated relatively equal loading. (B) FAK overexpression increased soft agar colonies by nearly 2.5-fold (*= *p* <0.05 vs. CON). (C) FAK overexpression increased SK-N-SH cell viability after 96 h (*= *p* <0.05 vs. CON). (D) Immunoblotting demonstrated transient FAK silencing (siFAK) in BE(2)-C cells. siFAK decreased phosphorylated protein levels of AKT and ERK1/2. β-actin demonstrated relatively equal loading. (E) siFAK inhibited colony formation by 35% compared to control (siNTC) (*= *p* <0.05 vs. siNTC). (F) siFAK significantly inhibited BE(2)-C cell viability after 96 h (*= *p* <0.05 vs. siNTC). (G) Immunoblotting demonstrated stable FAK silencing (shFAK) in BE(2)-C cells. β-actin demonstrated relatively equal loading. (H) Representative gross images of tumor from mice and H&E staining of liver sections (200× magnification, 50 μm bar) (I) Spleen and liver weight relative to body weight (*n* = 6 mice in each group; **= *p* <0.005 vs. shCON; Mann Whitney test)

In order to examine the critical role of FAK on neuroblastoma malignant potential, we transiently transfected SK-N-SH and BE(2)-C cells with FAK plasmid (Fig. 4A) and siRNA against FAK (siFAK) (Fig. 4D), respectively. To validate the effects of downstream signaling pathways on modulating FAK expression in each cell line, phosphorylated and total expressions of AKT and ERK1/2 were examined. Interestingly, modulation of FAK expression led to differential expression of p-AKT and p-ERK (Figs. 4A, D). FAK overexpression stimulated phosphorylation of AKT and ERK. Conversely, FAK silencing decreased p-AKT and p-ERK levels, hence suggesting a mechanism of FAK activation of PI3K and MEK pathways in neuroblastoma cells. We used soft agar colony assay to assess for anchorage-independent cell growth, which is a well-established indicator of tumorigenicity of cancer cells *in vitro* [17]. FAK overexpression showed an increased number of colonies by > 2.5-fold, and quite interestingly, it also resulted in formation of larger colonies (Fig. 4B). Furthermore, cell viability also increased over a time course with most significant increase at 96 h (Fig. 4C). In contrast, siFAK showed decreased number of colonies by 35%; siFAK resulted in fewer and smaller colonies when compared to siNTC (Fig. 4E). siFAK also significantly decreased BE(2)-C cell viability (Fig. 4F). To validate these findings *in vivo* and to further examine the metastatic potential of BE(2)-C cells stably-transfected with shRNA against either control (shCON) or FAK (shFAK), we performed intrasplenic injections of neuroblastoma cells in nude mice. Our laboratory established a murine model to investigate neuroblastoma metastasis, and reported that GRP-R silencing inhibited tumor growth and liver metastases *in vivo* [3]. The specificity of FAK silencing was demonstrated by immunoblotting (Fig. 4G). Six weeks after injections, tumor volumes of spleen and liver were examined. While shCON formed numerous large liver metastases, shFAK developed very few liver lesions (Fig. 4H). The average spleen and liver weight in the shFAK group was approximately 62% of the shCON group (mean value 0.08 for shCON vs. 0.05 for shFAK) (Fig. 4I). These results indicate that FAK silencing decreases malignant potential of neuroblastoma cells, thus providing further support for the importance of FAK as a regulator of neuroblastoma malignancy.

### FAK overexpression rescued GRP-R silencing-mediated inhibition of cell growth in vitro and in vivo

**Figure 5:**
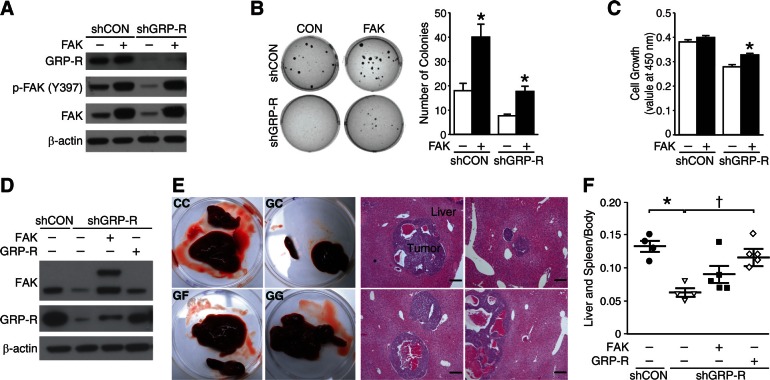
FAK overexpression in GRP-R silencing restores neuroblastoma growth (A) Immunoblotting confirmed transient FAK overexpression (pCMV6-PTK2) in shGRP-R. β-actin demonstrated relatively equal loading. (B) FAK overexpression increased soft agar colonies in shCON and shGRP-R when compared to controls of FAK overexpression (*= *p* <0.05 vs. FAK vector control). (C) FAK overexpression in shGRP-R restored cell viability (*= *p* <0.05 vs. FAK vector control). (D) Immunoblotting confirmed stable FAK overexpression (pCDH-FAK) in shGRP-R. β-actin demonstrated relatively equal loading. (E) Representative gross images of tumor from mice (shCON/pCDH = CC, shGRP-R/pCDH = GC, shGRP-R/pCDH-FAK = GF, shGRP-R/pCDH-GRP-R = GG) and H&E staining of liver sections (200× magnification, 50 μm bar) (F) Spleen and liver weight relative to body weight (*n* = 4-5 mice in each group; *= *p* <0.05 vs. CC, †= *p* <0.05 vs. GC; Mann Whitney test).

To assess whether FAK is a downstream target of GRP-R-mediated cell signaling, we next performed rescue experiments using a FAK plasmid in shGRP-R cells in order to test whether FAK overexpression recovers the inhibitory effect of shGRP-R. Immunoblotting showed knockdown of GRP-R and overexpression of FAK after transfections (Fig. 5A). As previously reported [3], shGRP-R exhibited significantly reduced number of soft agar colony formation. FAK overexpression resulted in increased colony formation in both shCON and shGRP-R cells (Fig. 5B). FAK overexpression in shGRP-R cells rescued their ability to form colonies similar in value to that of shCON. Correlative to soft agar colony formation, cell viability assays demonstrated restored cell growth after FAK overexpression in shGRP-R cells (Fig. 5C). Furthermore, to demonstrate these observations *in vivo*, we used a murine model after stably-transfecting BE(2)-C cells with a lentiviral system of FAK plasmid. Specificity of each protein expression was demonstrated by immunoblotting (Fig. 5D). BE(2)-C cells with GRP-R knockdown (GC) induced fewer metastatic lesions in the liver when compared to control (CC), and reintroducing GRP-R (GG) into cells rescued the inhibitory effect of GC (Fig. 5E). Although FAK overexpression in GC (GF) did not show statistically significant effects on liver metastasis, the mean weight of spleen and liver in these mice increased by ~1.5-fold of GC (mean value 0.06 for GC vs. 0.09 for GF) (Fig. 5F). These findings suggest that FAK plays a critical role in GRP-R-induced anchorage-independent growth. Also, as a downstream target of GRP-R, FAK expression, compensates, in part, for the inhibitory effect of GRP-R silencing in neuroblastoma. Taken together, our results demonstrate that FAK is an important mediator in the GRP/GRP-R signaling pathway.

### FAK inhibitor reduced BBS induced-tumor growth and metastasis in vitro and in vivo

**Figure 6:**
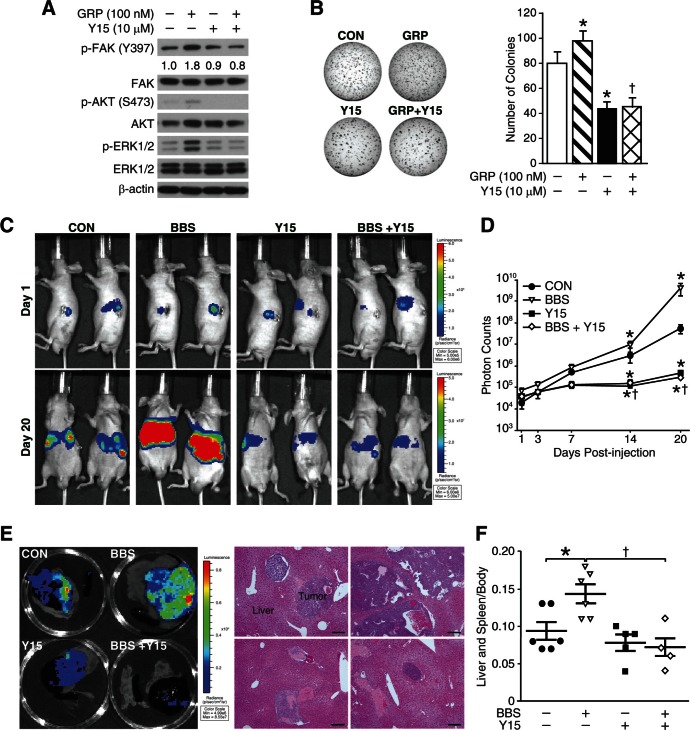
Y15, a FAK inhibitor blocks BBS-induced neuroblastoma growth and metastasis (A) Immunoblotting confirmed that GRP-induced FAK activation in BE(2)-C cells was blocked by pre-treatment of Y15 (10 μM) for 30 min. Y15 treatment attenuated GRP-induced activation of AKT and ERK1/2. (B) GRP (100 nM) increased colony formation by ~1.2-fold whereas Y15 (10 μM) inhibited it by 45% compared to control; similarly Y15 also inhibited GRP-induced colony formation (*= *p* <0.05 vs. CON, †= *p* <0.05 vs. GRP). GRP and Y15 were added into serum media on solidified soft agar. (C) Bioluminescence images showing luciferase-expressing BE(2)-C cells in an intrasplenic injection murine model. Pseudocolor images from each group were adjusted to the same threshold of the day. At day 1, mice were imaged on their left side to detect relatively equal intensity of luminescent signal of spleen. At day 20, BBS (20 μg/kg/i.p./t.i.d.) promoted tumor growth and liver metastases, whereas Y15 (30 mg/kg/i.p./day) showed the opposite effect, and further inhibited BBS-induced tumor growth and liver metastases. (D) Quantitative values of bioluminescence were measured as photon counts. Results were given as the mean ± SEM (*n* = 5-6 mice in each group; *= *p* <0.05 vs. CON, †= *p* <0.05 vs. BBS). (E) Representative bioluminescence images of tumor tissue extracted from the mice of each group and representative images of H&E staining to confirm metastatic foci of neuroblastoma cells in liver lesions (200× magnification, 50 μm bar) (F) Spleen and liver weight relative to body weight (*n* = 5-6 mice in each group; *= *p* <0.05 vs. CON, †= *p* <0.05 vs. BBS; Mann Whitney test).

Previously, we have reported that BBS, an amphibian equivalent of GRP, promotes neuroblastoma tumor growth *in vivo*, and is an important stimulator of angiogenic pathway [18, 19]. Additionally, Y15 compound, a FAK-specific inhibitor, has shown to decrease neuroblastoma growth *in vivo* [20, 21]. In this study, we wanted to determine whether FAK inhibition could block BBS-induced tumor growth and metastases *in vivo*. First of all, we found that pre-treatment with Y15 compound (10 μM) for 30 min failed to induce FAK activation at Y397 followed by GRP for 5 min after overnight serum starvation in BE(2)-C cells *in vitro* (Fig. 6A). GRP alone induced activation of AKT and ERK1/2, whereas Y15 treatment attenuated GRP-induced increases in p-AKT and p-ERK (Fig. 6A). Interestingly, the Y15 treatment also significantly inhibited GRP-induced colony formation in soft agar by > 2-fold (Fig. 6B). Based on these findings, 1 × 10^6^ of luciferase-expressing BE(2)-C cells were injected into spleen in athymic nude mice. After three days, they were randomized into four groups: control (PBS vehicle), BBS (20 μg/kg/i.p./t.i.d.), Y15 (30 mg/kg/i.p./day), and BBS plus Y15. Bioluminescence imaging system was used to monitor for primary tumor growth in spleen as well as liver metastasis. As shown in Figure 6C, signals exhibited relatively equal intensities on day 1; however, by day 20, BBS significantly increased primary splenic tumor growth as well as liver metastases while a combination treatment with Y15 showed remarkable reduction in tumor growth and metastases. The inhibitory effects of Y15 in BBS-induced tumor growth were confirmed by luciferase activity of luciferase-expressing BE(2)-C cells (Fig. 6C-E) as well as by spleen and liver weight (Fig. 6F). Differences in luciferase activity at day 20 correlated with tumor weights in the liver and spleen. BBS increased luciferase activity by > 80-fold as compared to controls (mean activity 5.30 × 10^7^ photons/s for controls vs. 4.27 × 10^9^ photons/s for BBS) whereas Y15 decreased the activity by < 110-fold as compared to controls (mean activity 5.30 × 10^7^ photons/s for controls vs. 4.78 × 10^5^ photons/s for Y15). More importantly, BBS plus Y15 combination significantly reduced luciferase activity when compared to BBS alone (mean activity 4.27 × 10^9^ photons/s for BBS vs. 2.65 × 10^5^ photons/s for BBS plus Y15). Thus, our *in vivo* data corroborate *in vitro* findings, and further suggest that FAK is an important regulator in GRP/GRP-R signaling pathway and tumor metastasis in neuroblastoma.

## DISCUSSION

We previously demonstrated that GRP-R is overexpressed in malignant, advanced-stage neuroblastomas, and that GRP-R silencing suppresses tumorigenesis and metastasis *in vivo* [3, 12]. However, the exact molecular mechanisms of GPR/GRP-R regulation of tumor progression are yet to be delineated. In this study, we provide compelling evidence from cell and animal studies that FAK is an important mediator of GRP/GRP-R signaling-induced neuroblastoma growth and metastasis. We investigated the relationship between GRP-R and FAK, as well as the effect of FAK silencing using transfected cells and/or a pharmacologic agent on tumor growth *in vitro* and *in vivo*. GRP/GRP-R regulated FAK activation and expression, and further inhibition of FAK repressed GRP/GRP-R signaling involved in neuroblastoma progression. These results suggested that FAK is a critical downstream target of GRP/GRP-R, and therefore may be a promising therapeutic target for malignant neuroblastomas.

GRP, the mammalian analogue of BBS, is a growth factor that promotes cell proliferation in cancer cells [22, 23]. Specifically, GRP induces activation of FAK at Y397 site, which is known to be critical for promoting both integrin- and/or growth-factor-stimulated cell migration, and is also a high-affinity binding site for Src homology 2 (SH2) of the Src family kinases [24]. These known associations create the possibility for interaction with a number of different signaling and adaptor proteins. In the present study, we found that GRP/GRP-R signaling regulates FAK activation and expression. We also found that GRP-R expression correlates with integrin expressions. Thus, our findings further support published results that integrin and growth-factor receptor signaling can interact through either membrane-proximal clustering of the two receptor types or the activation of common downstream signaling pathways [25, 26].

Our findings corroborate the multiple tumorigenic functions of FAK, which are associated with various malignant and aggressive tumors, including ductal carcinomas of the breast [27], primary colorectal tumors and metastases [28], and endometrial carcinomas [29]. FAK has been used as a prognostic indicator as well as a marker for malignant transformation in breast carcinoma [30]. Additionally, FAK has also been established as a significant component in the BBS signaling pathways in prostate cancer [8, 9, 11] and described to play a critical role in GRP-mediated morphogenesis of colon cancer [14]. Glover *et al*. [8] showed that the FAK Y397 site is critical for GRP-induced morphogenesis in 293 HEK cells, and we have shown that BBS promotes tumor growth and angiogenesis in neuroblastoma *in vivo* [18, 19]. Here, we show that GRP or BBS stimulates neuroblastoma cell migration as well as liver metastases, further supporting a critical tumorigenic role of GRP or BBS in neuroblastoma. More importantly, our results, for the first time, show that FAK inhibition is critical to block the BBS-induced tumorigenesis and metastasis *in vivo*, and thus, further indicating FAK as an important therapeutic target in the treatment of neuroblastomas.

Y15 compound, 1,2,4,5-benzenetetraamine tetrahydrochloride, has been shown to block phosphorylation of FAK at Y397 in neuroblastoma cell lines as well as inhibit growth of *Mycn*-amplified neuroblastoma tumors *in vivo* [20]. In our study, we used *Mycn*-amplified I-type BE(2)-C and *Mycn* single copy N-type SK-N-SH neuroblastoma cell lines. Since, *Mycn* regulates FAK expression at the promoter level in neuroblastoma cells [31], we speculated that GRP-R signaling would affect FAK by regulating *Mycn*. This is supported by the results that showed reduced level of FAK mRNA in GRP-R silenced BE(2)-C cells. However, the molecular mechanism of GRP/GRP-R signaling regulation of *Mycn* and subsequently FAK in *Mycn*-amplified neuroblastoma cells or *Mycn* single copy neuroblastoma cells remain to be defined.

Tilghman *et al*. [32] noted that the loss of FAK resulted in marked changes in cell morphology and defects in leading edge formation. Similar to their findings, we also found that silencing of FAK by either cell transfection or chemical compound induced morphological changes analogous to the effects of silencing of GRP-R, and further inhibited colony formation. Hence, we infer that GRP-R and FAK are essential for regulating cell morphology, which can associate with the oncogenic properties such as highly migratory, anchorage-independent phenotype in neuroblastoma. Therefore, a better understanding of GRP-R and FAK regulation may not only be of biological significance, but it may also provide a molecular basis for potential clinical applications.

In conclusion, our study demonstrates that FAK is a crucial regulator of GRP/GRP-R signaling in neuroblastoma. GRP/GRP-R regulates neuroblastoma cell growth, transformation and migration by correlative regulation of FAK. Furthermore, our results suggest that targeting FAK can inhibit GRP/GRP-R-mediated oncogenic properties. Moreover, our study demonstrates the roles of FAK in neuroblastoma tumorigenesis and metastasis *in vitro* and *in vivo*. These findings are clinically relevant because advanced-stage neuroblastoma is oftentimes refractory to current multi-modality treatment protocols, and effective novel therapeutic target(s) are highly desirable. Hence, a better understanding of the mechanisms involved in GRP-R/FAK-induced metastatic potential could provide insights into development of novel strategies in the treatment of aggressive, undifferentiated neuroblastoma.

## MATERIALS AND METHODS

### Antibodies and reagents

Primary antibodies used include GRP-R from Abcam, p-FAK, FAK from BD Biosciences, β-actin, Tubulin from Sigma-Aldrich, Mycn, p-AKT, AKT, p-ERK1/2, ERK1/2 from Cell Signaling Technology, integrin α3 and β1 from Santa Cruz Biotechnology, Alexa Fluor 568 and 488 from Molecular Probes. FAK (4.47) antibody for immunohistochemistry was from Millipore. GRP, BBS from Bachem, Y15 (C6H10N4·4ClH, 1,2,4,5-benzenetetraamine tetrahydrochloride) from Sigma-Aldrich were used. Agarose (SeaPlaque®) was from Cambrex Bio Science. Cell Counting Kit-8 (CCK-8) was from Dojindo. Immunohistochemistry reagents were from Dako. FAK, integrin α3 and β1 siRNAs along with non-targeting scrambled sequences was from Dharmacon.

### Cell culture and transfection

Plasmids for GRP-R overexpression and silencing have been described [2], and pCMV6-PTK2 from OriGene was used for transient FAK overexpression. shFAK and pCDH-FAK plasmids for *in vivo* study were kindly provided by Dr. S.T. Lim (University of South Alabama). pMSCV-LucSh, which contains a luciferase and zeocin-resistance fusion gene was also kindly provided by Dr. Andrew M. Davidoff (St. Jude Children's Research Hospital). Stably-transfected cells for GRP-R were established by selection with G418 at 300 μg/ml and/or zeocin at 50 μg/ml for 2 weeks as described [3]. Stable populations of shFAK cells were obtained by lentiviral infection and puromycin selection at 2.5 μg/ml. Luciferase-expressing cells were established by selection with zeocin at 50 μg/ml.

### Quantitative real-time PCR (QRT-PCR)

Total RNA was isolated using the RNAqueous™ (Ambion). Isolated RNA (1 μg) was used to synthesize cDNA using the High-Capacity cDNA Reverse Transcription Kit (Applied Biosystems). Primers were designed to amplify a 158-bp FAK fragment (BC028733.2): forward primer 5'-TTATTGGCCACTGTGGA TGA-3'; reverse primer 5'-TACTCTTGCTGGAGGCTGGT-3'. GRP-R and Glyceraldehyde 3-phosphate dehydrogenase (GAPDH) primers were the same as published [33]. QRT-PCR was performed in the CFX96™ Real-Time PCR Detection Systems using SsoFast™ EvaGreen Supermix (Bio-Rad).

### Immunofluorescence

Cells were fixed with 4% paraformaldehyde for 20 min at room temperature (RT), permeabilized with 0.1% Triton X-100 for 15 min and blocked with 1% BSA/PBS for 30 min. Cells were incubated with primary antibodies (1:100) for 1 h at RT, washed five times with PBS and then incubated with secondary antibodies (1:200) for 30 min at RT. The nuclei were counterstained with Vectashield mounting medium containing DAPI (Vector Laboratories). The immunofluorescence of FAK was observed under a fluorescent microscope (Nikon Eclipse E600).

### Immunohistochemistry

Tissues were fixed in formalin for 3 days and embedded in paraffin wax. Paraffin-embedded sections (5 μm) were deparaffinized in three xylene washes followed by a graded alcohol series, antigen retrieval performed with 10 mM sodium citrate buffer, and then blocked with blocking solution for 1 h at RT. Sections were incubated with primary antibodies overnight at 4°C, washed with PBS, incubated with secondary antibodies for 30 min at RT, and developed with DAB reagent. All sections were counterstained with hematoxylin, and then dehydrated with ethanol and xylene. Coverslips were mounted and slides observed by light microscopy.

### Immunoblotting

Whole cell lysates were collected using cell lysis buffer (20 mM Tris, 150 mM NaCl, 1 mM EDTA, 1 mM EGTA, 0.1% SDS, 1% sodium deoxycholate, 1% Triton X-100, aprotinin, leupeptin, and 1 mM sodium orthovanadate) supplemented with proteinase inhibitors (Roche). PMSF (1 mM) was added immediately prior to use. Protein (30 μg) was run on a SDS-PAGE gel, transferred onto a PVDF membrane, and probed with antibodies. Blots were developed using an enhanced chemiluminescence system (Amersham Biosciences). ImageJ (NIH) was used for densitometry.

### Cell viability

Cells were seeded at a density of 3 × 10^3^ cells in a 96-well plate and grown for up to 4 days after transfection. Cell numbers were assessed using CCK-8 daily. Assay was performed in triplicates and the values, corresponding to the number of viable cells, were read at OD450 with the EL808 Ultra Microplate Reader (Bio Tek Instrument).

### Colony formation

Cells were trypsinized and resuspended in media containing 0.4% agarose and 7.5% FBS and then overlaid onto a bottom layer of solidified 0.8% agarose in 5% serum media. SK-N-SH and BE(2)-C were plated at concentrations of 5'10^3^ cells/well and 3'10^3^ cells/well of a 12-well plate and incubated for 5 and 3 weeks, respectively. Colonies were stained with 0.05% Crystal Violet, photographed and quantified.

### Migration assay

Transwell filters (8 μm; Corning) were coated on the lower side with 5 μg/ml collagen type I (BD Biosciences) overnight and then blocked with 2.5% BSA/PBS for 1 h. 1 × 10^5^ cells in serum-free media added to the upper and incubated for 4 h. Cells were fixed with 4% paraformaldehyde, stained with DAPI and counted. Assay was performed in duplicates, and counting was from five randomly selected microscopic fields (200× magnification).

### In vivo assay

Male athymic nude mice (4–6 weeks old) were maintained as described [18]. All studies were approved by the Institutional Animal Care and Use Committee and were conducted in accordance with guidelines issued by the NIH. BE(2)-C cells were transfected with luciferase alone or stably-transfected with plasmids (shCON, shGRP-R, shFAK and/or pCDH, pCDH-FAK). 1 × 10^6^ cells of 50 μl of HBSS were intrasplenically injected as described [3]. Mice were treated daily with 50 μl of control vehicle (PBS) or Y15 (30 mg/kg/day) and/or BBS (20 μg/kg/t.i.d.). Dosages were chosen based on previous results [18, 21]. Tumor growth was observed by measuring luciferase signal with bioluminescence imaging system (IVIS Lumina II, Xenogen, Caliper Life Sciences) and body weight was measured weekly. Mice were injected with D-luciferin (OZ Biosciences) subcutaneously (1 mg/mouse in 100 μl of HBSS) before being anesthetized with isofluorane. Measurement of total flux (photons/sec) of the emitted light reflects the relative number of viable cells in the tumor. Data were analyzed using Xenogen Living Image software (version 4.1). At sacrifice, spleens and livers were excised, weighed and fixed in formalin for further analyses.

### Statistical analysis

*In vitro* data represent the means ± SD. Statistical analyses were performed using a Student's paired *t* test. *In vivo* experiments were analyzed as described [3, 18]. Data represent the means ± SEM. Body weight was analyzed using one-way ANOVA or Mann Whitney test with repeated measures on time. *p* value of <0.05 was considered statistically significant.

## Supplementary Figures


