# Supplementary material for: FAK is a Critical Regulator of Neuroblastoma Liver Metastasis
Source: Oncotarget. 2012 Nov 16;3(12):1576–87. doi: 10.18632/oncotarget.732 (PMC3681496; doi:10.18632/oncotarget.732)
Supplement: Supplementary file 1 [file oncotarget-03-1576-s001.pdf]

FAK IS A CRITICAL REGULATOR OF NEUROBLASTOMA LIVER METASTASIS - Lee et al

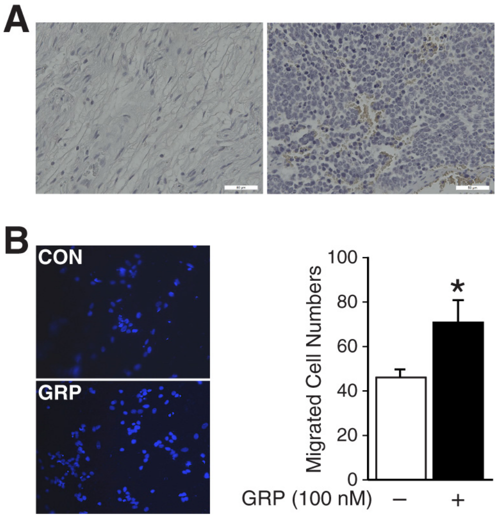

Figure S1: (A) Negative controls of histological section from human ganglioneuroma (left, secondary mouse antibody) and undifferentiated neuroblastoma (right, secondary rabbit antibody) by immunohistochemistry (50 µm bar indicated). (B) GRP (100 nM) treatment in bottom wells of transwell plate induced BE(2)-C cell migration. Values are expressed by counting DAPI staining of migrated cell numbers (200× magnification) (*= $p < 0.05$ vs. CON).
